# Effective antitumor peptide vaccines can induce severe autoimmune pathology

**DOI:** 10.18632/oncotarget.19688

**Published:** 2017-07-29

**Authors:** Hussein Sultan, Jimena Trillo-Tinoco, Paulo Rodriguez, Esteban Celis

**Affiliations:** ^1^ Cancer Immunology, Immunotherapy and Tolerance Program, Georgia Cancer Center, Augusta University, Augusta, GA, USA; ^2^ Biochemistry and Cancer Biology Department, Georgia Cancer Center, Augusta University, Augusta, GA, USA

**Keywords:** peptide vaccine, anti-tumor effect, diabetes, IL-2 complex, anti-CD40

## Abstract

Immunotherapy has shown a tremendous success in treating cancer. Unfortunately, this success is frequently associated with severe autoimmune pathology. In this study, we used the transgenic RIP-gp mouse model to assess the antitumor therapeutic benefit of peptide vaccination while evaluating the possible associated autoimmune pathology. We report that palmitoylated gp33-41 peptide and poly-IC adjuvant vaccine (BiVax) generated ∼ 5-10 % of antigen specific T cell responses in wild type and supposedly immune tolerant RIP-gp mice. Boosting with BiVax in combination with αCD40 antibody (TriVax) or BiVax in combination with IL-2/αIL-2 antibody complexes (IL2Cx) significantly increased the immune responses (∼30-50%). Interestingly, although both boosts were equally effective in generating vast T cell responses, BiVax/IL2Cx showed better control of tumor growth than TriVax. However, this effect was associated with high incidence of diabetes in an antigen and CD8 dependent fashion. T cell responses generated by BiVax/IL2Cx, but not those generated by TriVax were highly resistant to PD-1/PD-L1 inhibitory signals. Nevertheless, PD-1 blockade enhanced the ability of TriVax to control tumor growth but increased the incidence of diabetes. Finally, we show that severe autoimmunity by BiVax/IL2Cx was prevented while preserving outstanding antitumor responses by utilizing a tumor antigen not expressed in the pancreas. Our data provides a clear evidence that peptide based vaccines can expand vast endogenous T cell responses which effectively control tumor growth but with high potential of autoimmune pathology.

## INTRODUCTION

Cancer immunotherapy has been shown to be effective in treating many cancer types. Immunotherapy is gradually replacing many traditional cancer treatments (chemotherapy and radiation) as a first line therapy. Specifically, blockades of the checkpoint inhibitors, programmed cell death-1 (PD-1) and cytotoxic T-lymphocyte-associated antigen 4 (CTLA-4) have demonstrated significant antitumor efficacy in the clinic [1–3]. Unfortunately, the success of checkpoint blockade has frequently been associated with serious autoimmune reactions [4, 5]. In addition, the efficacy of checkpoint blockade appears to rely on the existence of antitumor T cells, which in many instances are non-existent or insufficient to achieve an antitumor effect. In view of this, it is not surprising that only a limited number of patients show objective responses to checkpoint blockade therapy. Thus, we advocate that therapeutic vaccines capable of generating substantial numbers of antitumor T cells will enhance the overall efficacy of checkpoint blockade therapy.

For some time, our group has been developing vaccination strategies capable of stimulating and expanding substantial numbers of tumor-reactive T cells and recently we were successful with a peptide vaccine where 20-60 % of total CD8 T cells were shown to be antigen specific and capable of limiting tumor growth in mice. One of these vaccines (TriVax) contained 3 components, a minimal CD8 T cell peptide epitope, poly-IC adjuvant, and αCD40 costimulatory mAb [6–8]. More recently, we reported that another vaccine with just 2 components (BiVax) using an amphiphilic peptide construct and poly-IC also generated substantial T cell responses [9]. The therapeutic antitumor responses of TriVax and BiVax against established B16 melanomas were greatly improved with the concurrent administration of either αPD-L1 mAb or with IL-2/αIL-2 mAb complexes (IL2Cx) [9, 10]. In these vaccination studies, we used CD8 peptide epitopes from melanosomal differentiation antigens (Trp1 and Trp2) and we observed that the antitumor efficacy correlated with the occurrence of widespread vitiligo, an autoimmune reaction that is not considered life threatening. These findings indicated that these vaccines can overcome immune tolerance, but the question arises whether they could generate more severe autoimmune pathology if a peptide epitope was expressed in a vital organ. To examine this possibility, we used the well-characterized RIP-gp transgenic mouse model that expresses a viral glycoprotein (LCMV-gp) in the pancreas [11]. The goal of the present study was to evaluate the ability of the optimized peptide vaccines to induce therapeutic antitumor effects in RIP-gp mice and examine the possibility of triggering severe autoimmune pathology in the form of diabetes. The results in this model system show that vaccines containing immunodominant CD8 T cell epitopes from LCMV-gp can overcome immune tolerance and generate massive T cell responses in RIP-gp mice. However, to achieve optimal therapeutic antitumor effects the vaccines had to be administered in combination with either IL2Cx or PD-1 blockade, which resulted in an increased incidence of diabetes. On the other hand, peptide vaccination utilizing a tumor specific antigen was effective against established tumors without inducing autoimmune pathology.

## RESULTS

### Optimization of an LCMVgp33-41 peptide vaccination strategy

**Figure 1:**
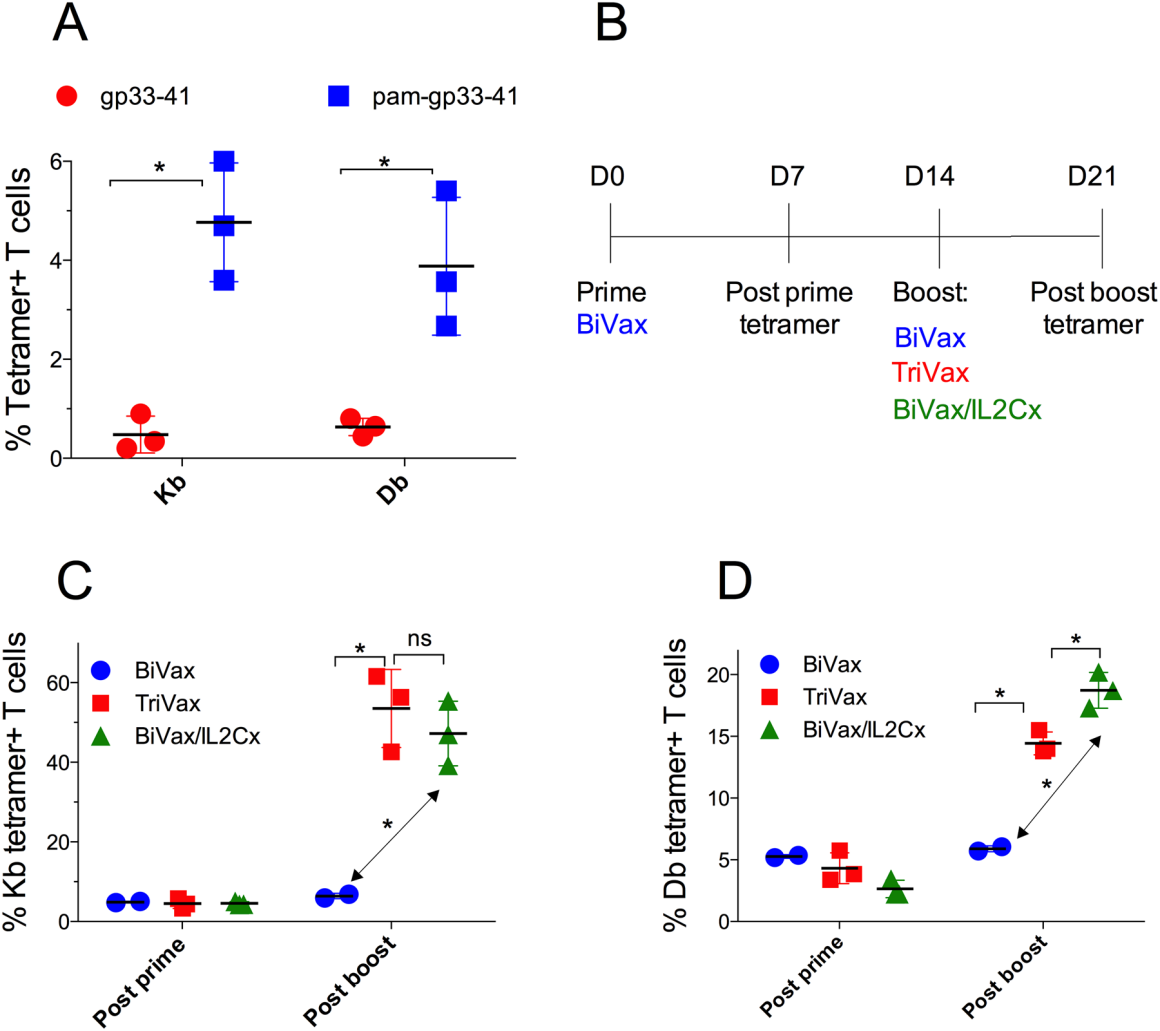
Optimization of gp33-41 vaccination strategy in WT mice **(A)** WT mice were primed with equimolar amounts of the pam-gp33-41 (Pam2-KMFVTAPDNLGYM) or minimal gp33-41 peptide (KAVYNFATM) and 50 μg poly-IC and 7 days later the immune responses were evaluated by tetramer staining in blood. **(B)** Diagrammatical representation of the vaccination protocol for panels C and D. **(C, D)** WT mice were primed with pam-gp33-41 BiVax and 14 days later they were boosted with pam-BiVax, pam-BiVax/IL2Cx or pam-TriVax. The percentages of Kb (B) and Db (C) tetramer+ CD8 T cells in blood after prime and boost are shown. Results are presented for individual mice (each symbol) with the mean ± SD for each group. (*p<0.05, ns: not significant).

Our previous studies using several peptide vaccination strategies in mice have shown that the combined use of poly-IC as an adjuvant, immune costimulation (αCD40 mAb or IL2Cx), modified peptides (amphiphilic constructs) and systemic mode of administration (i.v. injections) can result in the rapid induction of massive CD8 T cell responses that lead to significant therapeutic antitumor effects [6, 8, 10]. Using peptide epitopes from melanosomal differentiation antigens (Trp1 and Trp2) we observed that these vaccines could overcome immune tolerance and in the long run could generate autoimmunity in the form of widespread vitiligo [6]. Being cognizant that effective antitumor immunotherapy can lead to severe autoimmune pathology [5], we undertook the present studies to evaluate some of the more severe potential autoimmune adverse effects of antitumor T cell vaccination. We selected the RIP-gp transgenic mouse model expressing the LCMV glycoprotein in the pancreas to determine whether peptide vaccination in these mice could lead to effective antitumor CD8 T cell responses that could potentially lead to diabetes. First, we evaluated several peptide vaccination options in wild type (WT) B6 mice using peptide LCMV gp33-41 which contains 2 different CD8 T cell epitopes: one (KAVYNFATM) inducing T cells restricted by the H-2Db (Db) allele and another (AVYNFATM) generating responses restricted by H-2Kb (Kb) [12]. We tested the ability of vaccines containing either the minimal gp33-41 peptide or a palmitoylated amphiphilic peptide construct (pam-gp33-41) in combination with poly-IC (BiVax) to induce antigen-specific CD8 T cell responses. As shown in Figure 1A, a single i.v. BiVax immunization with pam-gp33-41 induced a substantially higher CD8 T cell responses to both the Kb and Db epitopes as compared to the minimal gp33-41 peptide. Thus, for all future experiments we solely used pam-gp33-41 as the immunogen. Next, we evaluated the effect of a booster vaccination and whether the addition of costimulatory αCD40 mAb (TriVax) or IL-2/αIL-2 mAb immune complexes (BiVax/IL2Cx) to the boost would further augment the CD8 T cell responses (protocol shown in Figure 1B). A dramatic enhancement in the levels of antigen-specific CD8 T cells was observed when the boosts were administered with either TriVax or BiVax/IL2Cx as compared to boosting with BiVax alone (Figure 1C, 1D). Although the levels of Kb and Db restricted antigen-reactive CD8 T cell responses were similar after the vaccine prime (∼5%), substantially higher frequencies of the Kb restricted CD8 T cells were observed after the boost with TriVax or BiVax/IL2Cx (Figure 1C, ∼50%) as compared to the Db epitope responses (Figure 1D, ∼15%). Thus, the Kb response to gp33-41 peptide dominates over the Db response when pam-gp33-41 peptide was used as immunogen. Both TriVax and BiVax/IL2Cx boosts enhanced the levels of antigen-specific T cells to similar levels (< 5% difference).

### TriVax or BiVax/IL2Cx generate vast T cell responses in RIP-gp mice

**Figure 2:**
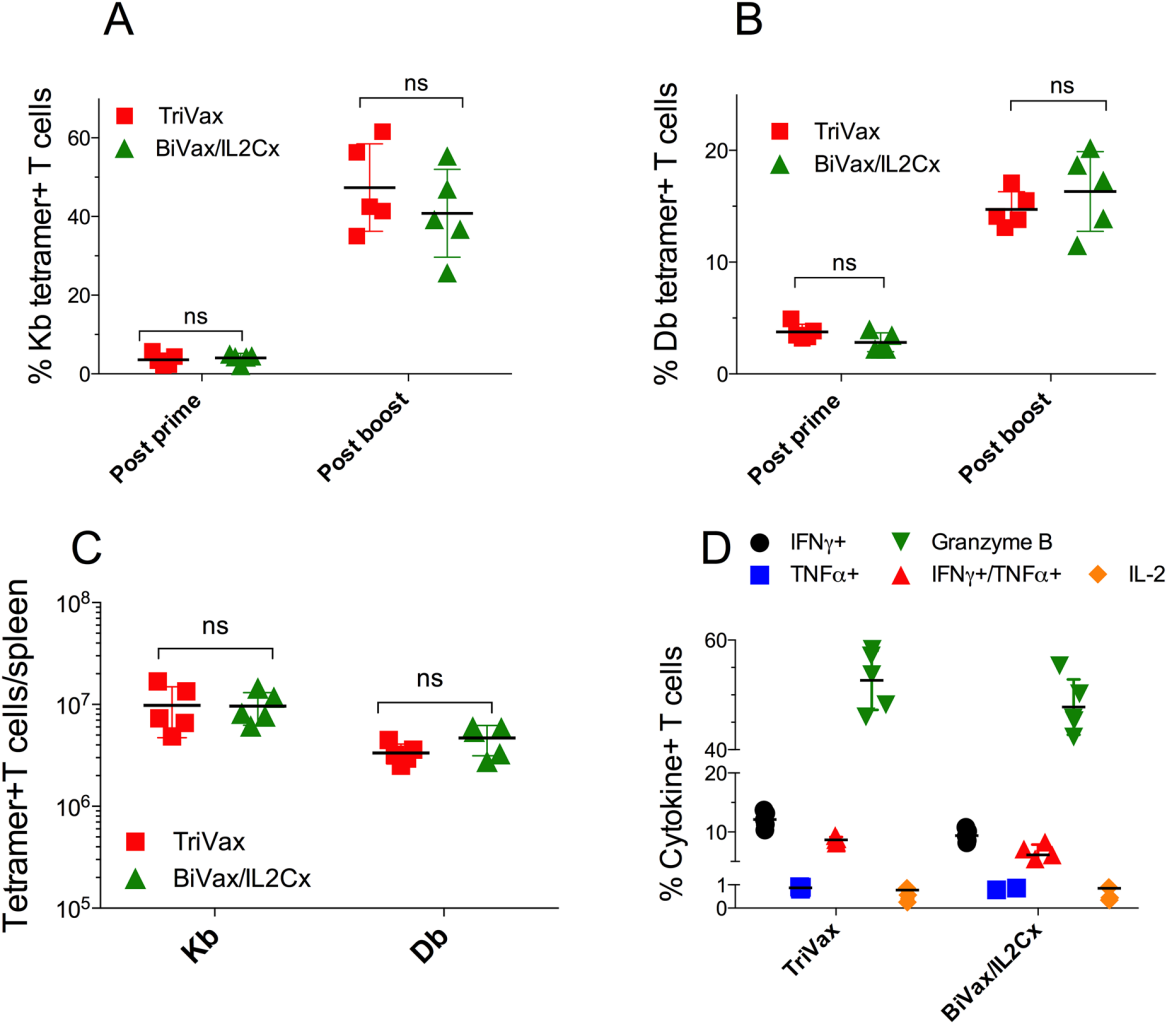
TriVax or BiVax/IL2Cx generate vast T cell responses in RIP-gp mice **(A, B)** RIP-gp mice were vaccinated with pam-gp33-41 BiVax and 14 days later they were boosted with TriVax or BiVax/IL2Cx. Percentage of Kb (A) and Db (B) tetramer+ CD8 T cells in the blood. **(C)** Total numbers of Kb and Db specific CD8 T cells in spleens after boost. **(D)** Splenocytes were stimulated *in vitro* with the minimal gp33-41 peptide in the presence of Golgiplug for 6 h and the production of IFNγ, TNFα, IFNγ/TNFα, IL-2 and granzyme B was assessed by intracellular staining. Results are presented for individual mice (each symbol) with the mean ± SD for each group. (ns: not significant).

**Figure 3:**
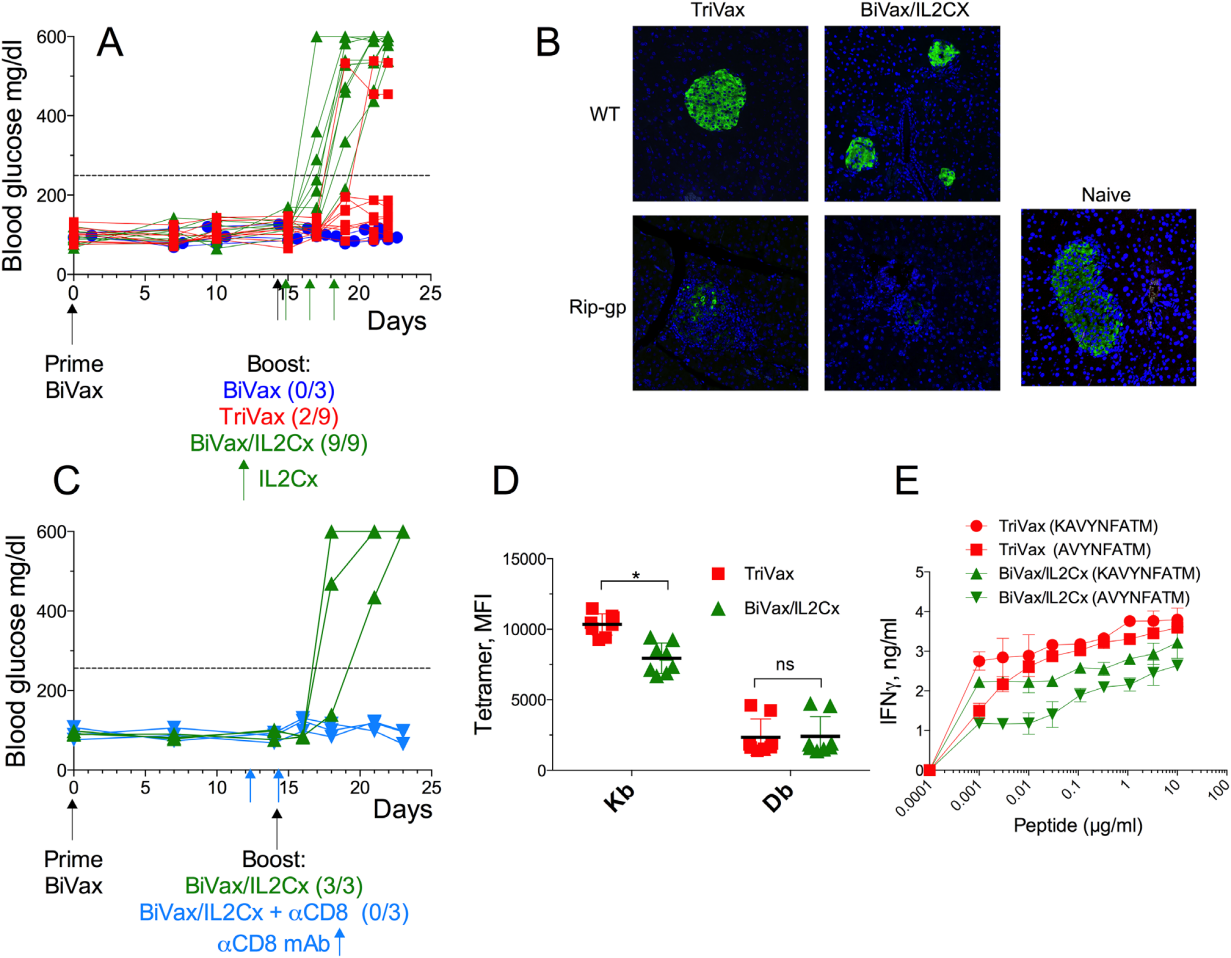
BiVax/IL2Cx but not BiVax alone or TriVax induces diabetes in Rip-gp mice **(A-E)** RIP-gp mice were vaccinated as described in Figure 1B. (A) Blood glucose levels in individual mice (each symbol) from at least 3 independent experiments. (B) Insulin staining in formalin fixed pancreatic tissues of WT and RIP-gp mice after TriVax or BiVax/IL2Cx boost was analyzed 8 days after the booster vaccination. (C) RIP-gp mice were primed with BiVax followed by BiVax/IL2Cx. Some mice received 500 μg of αCD8 mAb (i.p.) at days 12 and 14. Blood glucose levels were measured to assess diabetes. (D) Mean fluorescence intensity (MFI) of tetramer stains for Kb and Db specific cells in RIP-gp mice. Each symbol represents an individual mouse. (E) Purified CD8 T cells were incubated with serial dilutions of the minimal Db (KAVYNFATM) or Kb (AVYNFATM) peptides and 48 h later the production of IFNγ in the supernatants was assessed by ELISA. Dashed horizontal lines in A and C represents maximal normal blood glucose level. (*p<0.05, ns: not significant).

Next, we determined the ability of the optimized vaccination strategies to induce CD8 T cell responses in presumably immune tolerant RIP-gp mice. Both TriVax and BiVax/IL2Cx boosts generated equally strong CD8 T cell responses to both Kb (Figure 2A) and Db (Figure 2B) epitopes, which were only slightly lower to those observed in WT mice (Figure 1C, 1D). The magnitude of the overall response, as assessed by the total numbers of antigen-specific CD8 T cells in spleens of the RIP-gp mice, was remarkable (Figure 2C, ∼10 million/spleen) considering that a mouse spleen has ∼20 million CD8 T cells. The ability of the CD8 T cells to respond to antigen was evaluated *in vitro* by peptide stimulation followed by intracellular cytokine staining (ICS). CD8 T cells from TriVax or BiVax/IL2Cx boosted mice showed similar capacity to produce IFNγ, TNFα and granzyme B, but failed to produce IL-2 (Figure 2D, dot plot examples shown in Supplementary Figure 1). These results suggest that both vaccines have similar capacity to stimulate and expand vast numbers of functional self-antigen specific CD8 T cells in RIP-gp mice and presumably overcome any existing immune tolerance. In these experiments, 100% (9/9) of the RIP-gp mice that were boosted with BiVax/IL2Cx and 22% (2/9) of the TriVax boosted mice developed diabetes (Figure 3A). Mice that received a BiVax boost without IL2Cx or αCD40 mAb did not develop diabetes. Staining formalin fixed pancreas sections with anti-insulin antibody showed a decrease/loss of reactivity in the TriVax and BiVax/IL2Cx vaccinated RIP-gp mice as compared to non-vaccinated RIP-gp animals (Figure 3B). CD8 T cell depletion 2 days before the boost abrogated the ability of BiVax/IL2Cx to induce diabetes (Figure 3C) and mice vaccinated with an irrelevant peptide (pam-Ova257-264) with BiVax/IL2Cx, which generated a vast immune response (∼50% tetramer+ T cells in blood), did not develop diabetes (Supplementary Figure 2).

The superior capacity of the BiVax/IL2Cx boost to induce diabetes as compared to TriVax boost did not appear to be related to differences in the numbers of antigen-specific CD8 T cells induced by these vaccines (Figure 2C). Thus, the possibility existed that differences in the T cell receptor (TCR) affinity elicited by the 2 different boosters could be a factor in determining their autoimmune function [13, 14]. We compared the TCR affinity of the antigen specific cells after TriVax or BiVax/IL2Cx boosts in RIP-gp mice by 2 different methods. First, we evaluated the mean fluorescence intensity (MFI) staining using tetramers for the Kb and Db epitopes. The tetramer MFI for the Kb epitope was higher in the TriVax boosted mice as compared to the BiVax/IL2Cx boosted animals while no substantial differences were observed for the Db epitope (Figure 3D). The TCR affinity was also evaluated using peptide titration T cell stimulation assays. Again, T cells from the TriVax boosted mice exhibited a superior activity against the Kb epitope as compared to the T cells from the BiVax/IL2Cx vaccines (Figure 3E). Also, the responses to the Kb epitope were somewhat higher in the TriVax boosted mice as compared to the BiVax/IL2Cx group. Together these results suggest that the increased efficacy of the BiVax/IL2Cx CD8 T cell responses to induce diabetes was not simply due to an increase of the quantity or quality of the T cell response (*i.e.,* TCR avidity) as compared to the T cells generated in TriVax boosted mice.

### BiVax/IL2Cx generates T cells that resist PD-L1 inhibition

**Figure 4:**
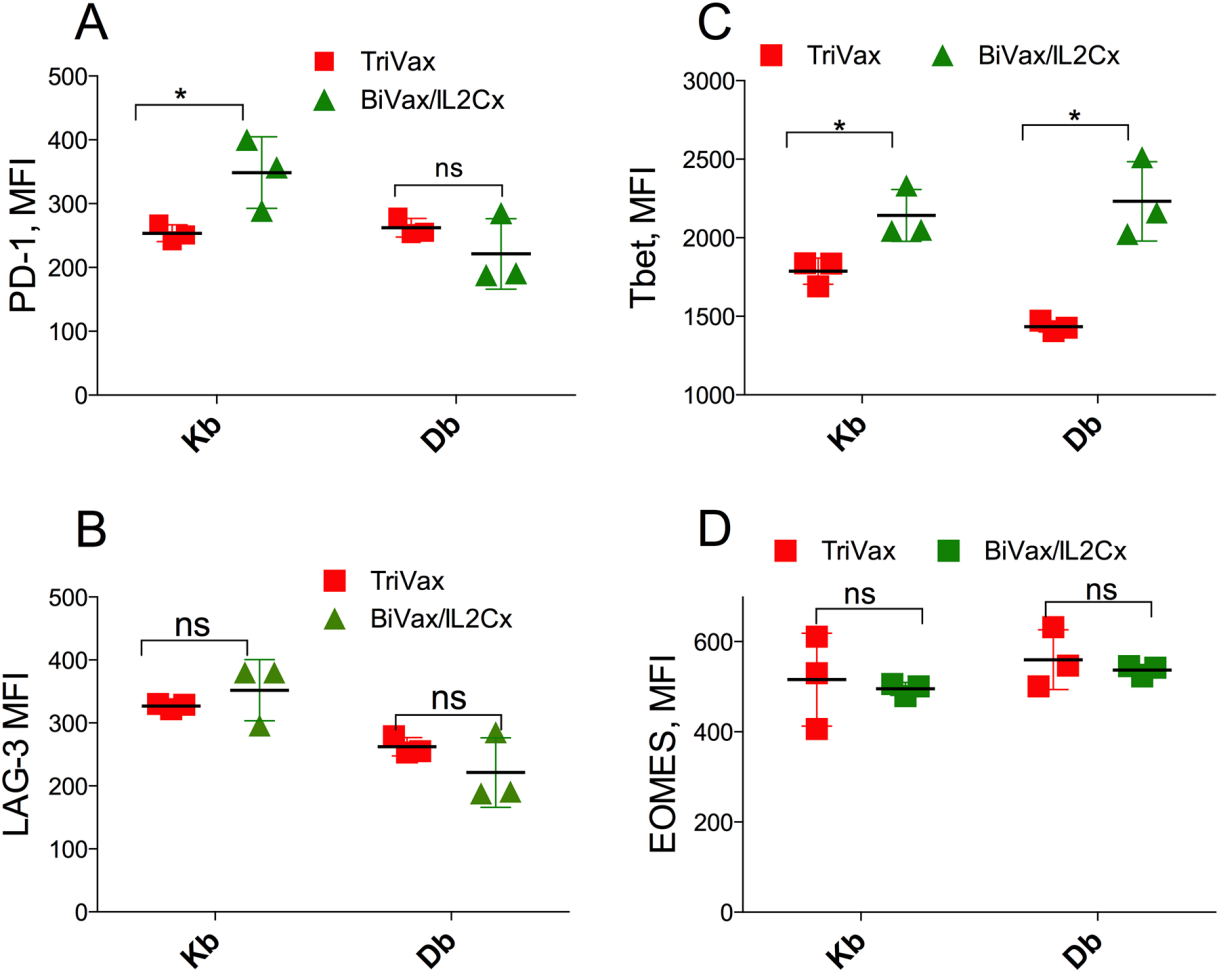
BiVax/IL2Cx generates T cell with relatively high Tbet expression **(A-D)** RIP-gp mice were vaccinated as in Figure 1B and spleens were collected 7 days after the boost. MFI of PD-1 (A) and LAG3 (B) expression on the surface of Kb and Db specific T cells are shown. MFI of Tbet (C) and Eomes (D) expression in Kb and Db specific T cells. Results are presented for individual mice (each symbol) with the mean ± SD for each group. (*p<0.05, ns: not significant).

We previously reported that IL2Cx administration after adoptive T cell transfer and TriVax immunization in mice bearing B16 melanoma tumors was able to overcome PD-1 inhibition resulting in tumor eradications [10]. These findings together with the above results suggested that BiVax/IL2Cx generates CD8 T cell responses that have the capacity to recognize self-antigen in the pancreas of RIP-gp mice and resist PD-1 inhibition signals leading to the onset of diabetes. Thus, we first determined whether the levels of the activation marker PD-1 expressed by the antigen-specific (tetramer positive) CD8 T cells from RIP-gp mice immunized with BiVax/IL2Cx were lower as compared to the T cells from TriVax boosted mice. However, mice immunized with BiVax/IL2Cx did not express lower levels of PD-1 as compared to the mice vaccinated with TriVax (Figure 4A, histogram plot examples shown in Supplementary Figure 3). Similar findings were observed when measuring the expression of LAG-3 (leukocyte activation gene-3), another T cell inhibitory receptor (Figure 4B, Supplementary Figure 3). It has been reported that Tbet expression enhances the ability of T cells to resist PD-1 inhibition of antigen specific T cells and sustain the effector responses in chronic infections [15–17] and that Tbet regulates autoimmune T cells that induce diabetes in mice [18, 19]. Accordingly, we observed that the expression level of Tbet in the antigen specific cells generated by BiVax/IL2Cx was slightly, but significantly higher compared to the TriVax group (Figure 4C, Supplementary Figure 3). On the other hand, no significant differences were evident in the expression levels of Eomes, another T-box transcription factor, which regulates T cell memory function (Figure 4D, Supplementary Figure 3). These data suggest that increased Tbet expression on T cells generated by BiVax/IL2Cx may contribute to enhance their ability to resist PD-1 inhibition signals.

**Figure 5:**
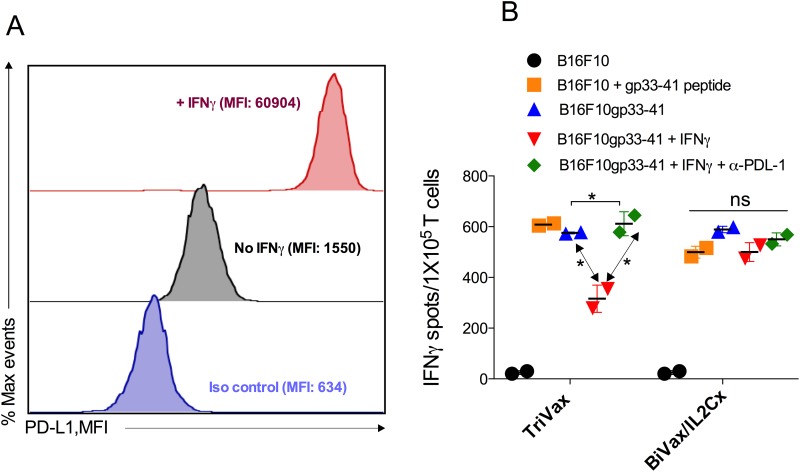
BiVax/IL2Cx generates T cell responses resistant to PD-L1 inhibition **(A)** Level of PD-L1 expression in B16F10gp33-41 cells (stimulated or not with IFNγ for 48 h). **(B)** 10^5^ purified CD8 T cells were incubated with untreated or IFNγ treated B16F10gp33-41 cells, in the presence or the absence of 10 μg αPD-L1, for 24 h and the numbers of IFNγ spots were enumerated by EliSpot. Results are presented as mean ± SD. (*p<0.05, ns: not significant). Results are presented for individual mice (each symbol) with the mean ± SD for each group. (*p<0.05, ns: not significant).

Next, we examined whether CD8 T cells from BiVax/IL2Cx would respond better to antigen stimulation in the presence of PD-1 inhibitory signals as compared to T cells from TriVax immunized mice. For these experiments, we utilized B16F10 melanoma cells transfected with a minigene encoding the LCMVgp33-41 epitope (B16F10gp33-41) [20], which upregulate surface PD-L1 expression after treatment with IFNγ (Figure 5A). CD8 T cells from both groups recognized B16F10gp33-41 cells to the same extent in an antigen-specific manner since untransfected B16F10 did not stimulate the cells, unless they were pulsed with peptide (Figure 5B). IFNγ-treated B16F10gp33-41 cells (PD-L1 high) were recognized to a lesser extent (∼50%) by the TriVax derived CD8 T cells as compared to the BiVax/IL2Cx generated T cells. Moreover, blocking PD-1/PD-L1 interactions with αPD-L1 mAb restored the capacity of TriVax generated CD8 T cells to recognize the IFNγ-treated B16F10gp33-41 cells but did not have any effect on the BiVax/IL2Cx induced T cells.

### TriVax/αPD-L1 combination induces diabetes in RIP-gp mice

**Figure 6:**
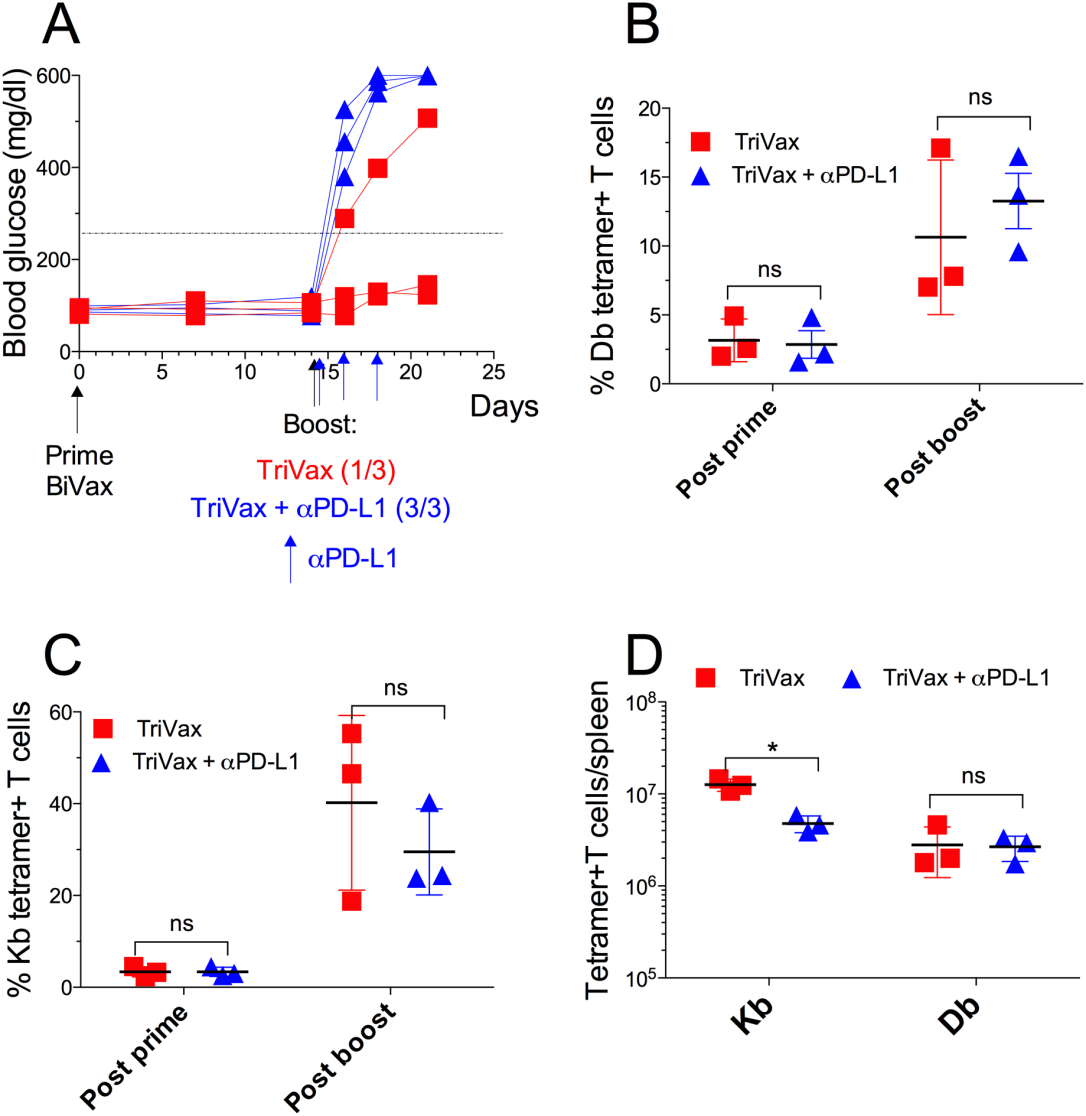
TriVax/αPD-L1 combination induces diabetes in RIP-gp mice RIP-gp mice were primed with pam-gp33-41 BiVax and 14 days later they were boosted with TriVax with or without αPD-L1 mAb (200 μg/mouse i.p. at days 14, 16 and 18). **(A)** Blood glucose levels in individual mice (each symbol) for each group. Percentages of Db **(B)** and Kb **(C)** tetramer+ CD8 T cells in the blood. **(D)** Absolute numbers of Kb and Db tetramer+ CD8 T cells in spleens. Results are presented for individual mice (each symbol) with the mean ± SD for each group. (ns: not significant).

The above findings indicate that the CD8 T cells from TriVax boosted mice are more susceptible to PD-1 inhibition than T cells from BiVax/IL2Cx vaccinated mice, which would explain their reduced ability to induce diabetes assuming that PD-1 checkpoint inhibition is present in the pancreas of the vaccinated RIP-gp mice. It has been shown that the costimulatory αCD40 mAb (a component of TriVax) increases the production IFNγ [21] enhancing PD-L1 expression in various tissues including pancreas [22]. Thus, we evaluated whether PD-1 blockade would augment the ability of a TriVax boost to induce diabetes in the RIP-gp mice. Indeed, concomitant administration of αPD-L1 mAb during TriVax boosts enhanced the incidence of diabetes (Figure 6A). The possibility that αPD-L1 mAb enhanced diabetes by increasing the magnitude of the T cell response was contemplated. However, since the administration of the αPD-L1 mAb did not increase the numbers of the antigen specific CD8 T cells (Figure 6B–6D), these results indicate that the effect of PD-1 blockade was through blocking inhibitory signals in the pancreas resulting in diabetes induction in the RIP-gp mice.

### Therapeutic antitumor efficacy of peptide vaccination

**Figure 7:**
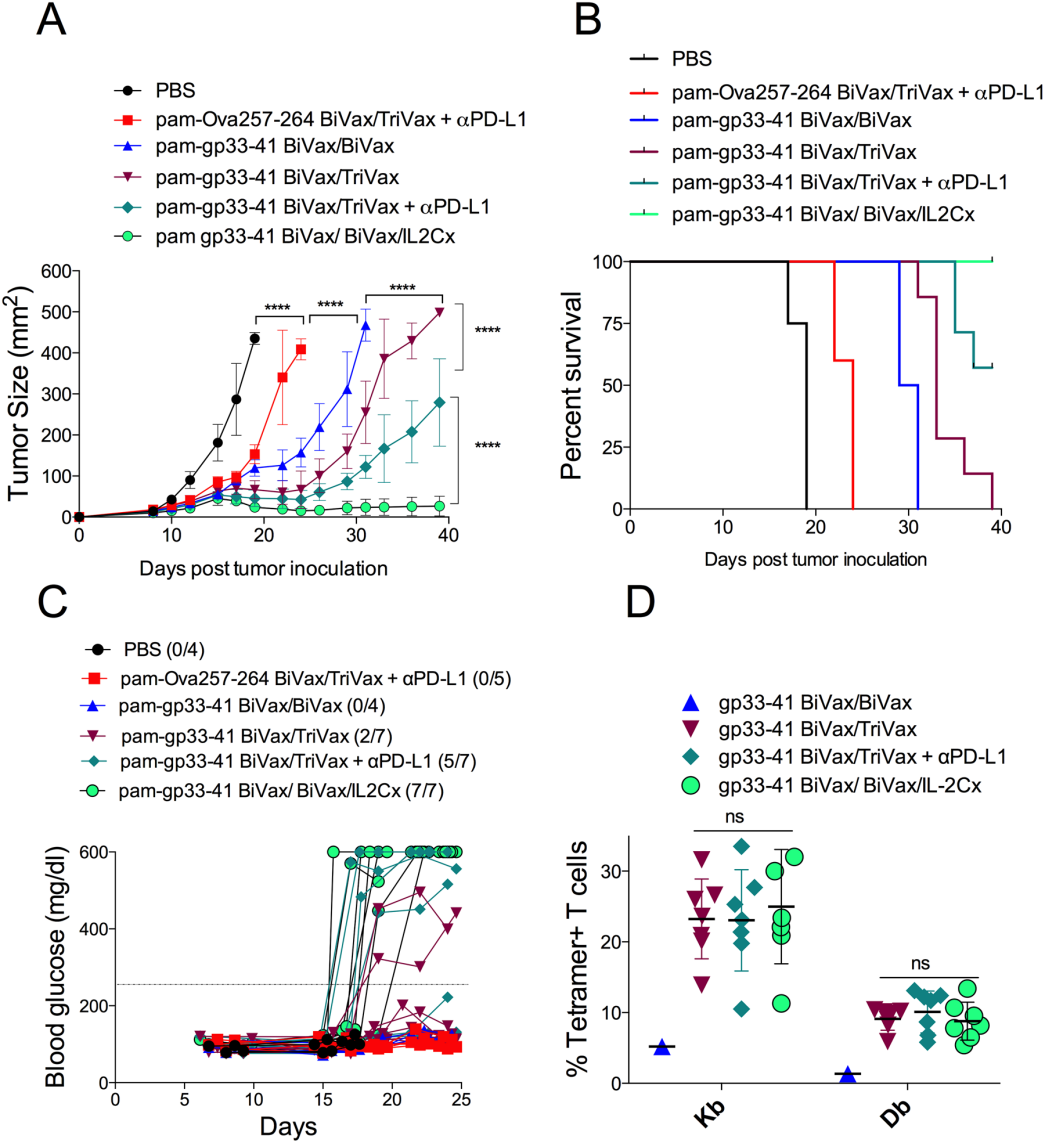
BiVax/IL2Cx combination show better therapeutic antitumor effects RIP-gp mice were inoculated s.c. with B16F10gp33-41 melanoma cells (3 × 10^5^ cells/mouse). After 8 days mice received pam-gp33-41 BiVax and 9 days later (17 days after tumor inoculation) they were boosted with either pam-gp33-41 BiVax, pam-gp33-41 TriVax, pam-gp33-41 TriVax+αPD-L1 or pam-gp33-41 BiVax/IL2Cx. αPD-L1 mAb was administered i.p. on days 17, 19 and 21. A control group was primed with pam-Ova257-264 BiVax and 9 days later boosted with pam-Ova257-264 TriVax+αPD-L1 mAb. **(A)** Mean tumor sizes and **(B)** overall survival of tumor-bearing mice. **(C)** Blood glucose levels in individual mice (each symbol) for each group. **(D)** Percentages of Kb and Db tetramer+ CD8 T cells for each individual mouse in the blood at day 30 after tumor inoculation. Results presented for individual mice (each symbol) with the mean ± SD for each group. (*p<0.05, ****p<0.0001, ns: not significant).

So far, our results indicate that both TriVax and BiVax/IL2Cx vaccination strategies were capable of overcoming immune tolerance to a self-antigen in tumor-free mice. Next, we investigated whether these vaccines could offer antitumor therapeutic benefit in a melanoma model. RIP-gp mice bearing 8-day established B16F10gp33-41 s.c. tumors received different vaccination options and tumor growth was monitored for 39 days. BiVax/IL2Cx was the most effective vaccination strategy allowing complete control of tumor growth throughout the duration of the experiment (Figure 7A, 7B). The second most effective vaccine was TriVax+αPD-L1 mAb, followed by the TriVax and BiVax boosts. TriVax vaccination with irrelevant peptide (pam-Ova257-264) in combination with αPD-L1 mAb showed some therapeutic benefit but significantly lower as compared to mice vaccinated with the pam-gp33-41 peptide. The degree of antitumor therapeutic benefit correlated with autoimmune pathology (Figure 7C) but not so much with the intensity of the CD8 T cell response (Figure 7D). Overall, these results demonstrate that peptide vaccination followed by either a BiVax/IL2Cx or a TriVax + αPD-L1 mAb boost can overcome immune tolerance and induce strong autoimmune antigen-specific CD8 T cells capable of providing therapeutic benefit against established tumors, but also responsible for causing severe autoimmune pathology.

**Figure 8:**
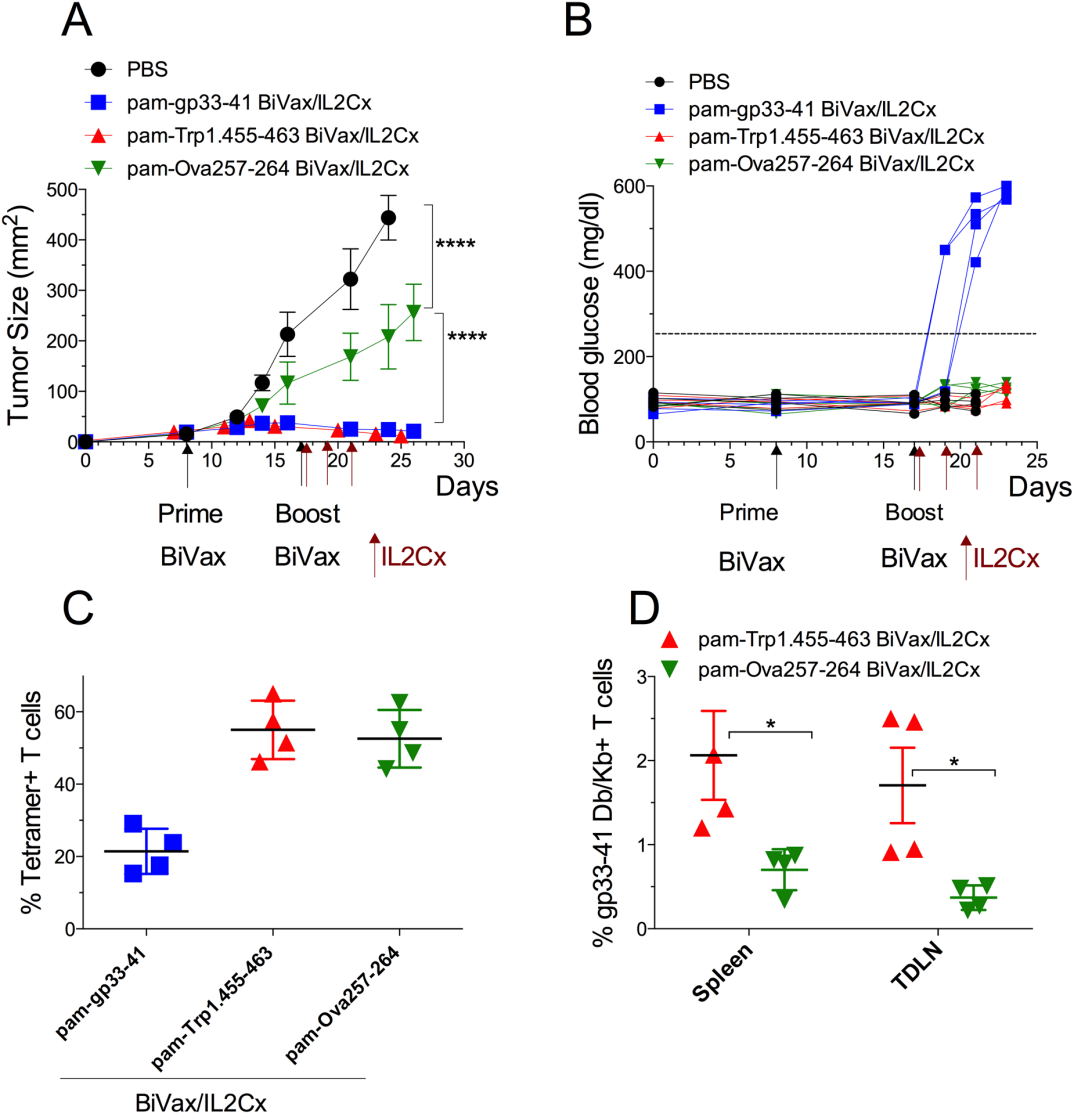
BiVax/IL2Cx utilizing tumor specific antigen controls tumor growth with limited autoimmune pathology RIP-gp mice were inoculated s.c. with B16F10gp33-41 melanoma cells (3 × 10^5^ cells/mouse) and 8 days later they received either pam-gp33-41 BiVax, pam-Trp1.455-463 BiVax or pam-Ova257-263 BiVax. Nine days later (17 days after tumor inoculation) mice were boosted with the same peptide plus IL2Cx (administered on days 17, 19 and 21). **(A)** Mean tumor sizes and **(B)** blood glucose levels in individual mice (each symbol) for each group. **(C)** Percentages of tetramer+ CD8 T cells in the blood at day 25 after tumor inoculation (Kb tetramer responses for gp33-41). **(D)** Percentage of gp33-41 Db/Kb+ T cells in spleen and tumor draining lymph nodes (TDLN) of Trp1 and Ova vaccinated mice. Results are presented for individual mice (each symbol) with the mean ± SD for each group. (*p<0.05, ****p<0.0001, ns: not significant).

Lastly, we used the RIP-gp mouse model to determine whether a BiVax/IL2Cx peptide vaccination strategy using a tumor epitope not expressed in the pancreas could result in the development of diabetes. The tumor epitope not expressed in the pancreas selected was Trp1.455-463, which is expressed in B16 melanoma and C57BL/6 mouse melanocytes, and generates strong CD8 T cell responses with BiVax [9]. RIP-gp mice with established B16F10gp33-41 s.c. tumors received BiVax prime followed by BiVax/IL2Cx boost using one of three different peptides: pam-gp33-41, pam-Trp1.455-463 or pam-Ova257-264. As with previous experiments, the mice that received the pam-gp33-41 vaccine had a remarkable antitumor response (Figure 8A) but all developed diabetes (Figure 8B). The mice that received the pam-Trp1.455-463 vaccine had an equal outstanding antitumor response, but none developed diabetes and the mice vaccinated with pam-Ova257-263 had a small, but significant antitumor response, without diabetes. Tetramer analysis of the T cell responses revealed that all peptides induced very strong antigen-specific CD8 T cell responses (Figure 8C). In the case of the mice vaccinated with pam-Trp1.455-463 a small (1-2%), but significant T cell response to the gp33-41 epitope was observed suggesting that the immune destruction of the B16F10gp33-41 tumor cells by the Trp1-reactive T cells may generate epitope spreading resulting in a new T cell response to a tumor antigen not present in the vaccine, but present in the pancreas. However, this response was not sufficiently strong to cause autoimmune pathology.

## DISCUSSION

In the past immunotherapy had been considered an alternative approach for treating cancer that could provide therapeutic benefit supposedly without the severe toxicities associated with conventional therapies (chemotherapy and irradiation). However, it has now become evident that effective anti-cancer immunotherapy is usually accompanied by toxicities related to autoimmunity. Under normal circumstances the immune system regulates responses against self-antigens to avoid autoimmune pathology and any manipulation to trigger antitumor responses by overriding immune tolerance increases the risk of autoimmunity. Here we used the transgenic RIP-gp mouse model developed by P. Ohashi [11] to assess the therapeutic benefit and associated autoimmune pathology of peptide vaccination against a tumor antigen that is also expressed in a vital organ (pancreas). The same group reported that peptide-based vaccines administered in combination with either αCD40 mAb, poly-IC, LPS or CpG generated some degree of T cell immune responses but failed to induce diabetes in these mice [23, 24]. Peptides administered together with LPS + αCD40 mAb (similar to TriVax) increased the level of the T cell response and caused pancreatic T cell infiltrates but did not produce diabetes. On the other hand, LCMV infection generated much stronger T cell responses and induced diabetes in 100% of the mice. The peptide vaccines used by Ohashi’s group were able to induce diabetes in the RIP-gp mice only when the LCMV-gp specific T cell precursor frequency was artificially increased by either adoptive transfer of T cells from TCR transgenic P14 mice or by cross-breeding RIP-gp mice with P14 TCR transgenic mice [24–27]. Thus, these findings indicate that the peptide vaccines tested in these studies [23, 24] were not sufficiently immunogenic to generate T cell responses of a magnitude similar to those generated by viral infections. On the other hand, Ohashi’s group reported that vaccinations consisting of *ex vivo* peptide-pulsed, TLR-activated dendritic cells (DCs) generated diabetes in 50-80% of RIP-gp mice suggesting that their peptide vaccines were not very efficient in delivering the vaccine components (peptide and TLR ligand) to the DCs *in vivo*. These studies utilized the minimal gp33-41 peptide, which in our hands was substantially less immunogenic as compared to the amphiphilic peptide construct pam-gp33-41 (Figure 1A).

We previously reported that amphiphilic peptides are substantially more immunogenic than the minimal peptide epitopes or conventional long peptides, because they self-assemble into nanoparticles that could resemble viruses and may be more efficiently captured by DCs [10, 28]. In general, we have observed that a second vaccine administration (booster) results in a sizable increase in the levels of antigen-specific T cells as compared to the levels obtained after the vaccination prime [10]. However, the responses obtained by pam-gp33-41 and poly-IC (BiVax) did not increase after the boost mice (Figure 1C, 1D). In contrast, the addition of αCD40 mAb (TriVax) or IL2Cx to the boost, considerably increased the magnitude of the T cell responses. Interestingly, although the levels of antigen-specific T cell responses generated by the 2 different boosts were similar (Figure 2), boosting with IL2Cx caused a much higher incidence of diabetes as compared to the αCD40 mAb boost (Figures 3A, 7C and 8B). Similarly, BiVax/IL2Cx boosting lead to more effective antitumor therapeutic effects as compared to the TriVax boosts (Figure 7A, 7B). These results indicate that the induction of autoimmunity (diabetes) and antitumor efficacy of the peptide vaccines do not solely depend on T cell numbers but may be derived from other inherent (qualitative) properties of the T cells. One possible explanation for the differences observed between the two different boost types could be that the T cells generated with TriVax boost are more susceptible to inhibition by CD4 Tregs (which are supposed to block autoimmune pathology) as compared to the T cells derived from the BiVax/IL2Cx boost. However, depletion of CD4 T cells did not increase the magnitude of the CD8 T cell response or the incidence of diabetes generated by the TriVax boost (Supplementary Figure 4). Another plausible explanation for the divergent results is that the T cells generated with BiVax/IL2Cx boost are more resistant to PD-1 inhibition as compared to those derived from the TriVax boost. PD-1/PD-L1 interactions play a role in preventing tissue destruction by self-reactive T cells and it has been reported that PD-1 blockade increases the predisposition of autoimmunity and diabetes induction in non-obese diabetic mice (NOD) mice [29]. While the levels of PD-1 expressed on the antigen-specific T cells were not significantly different between the two T cell populations (Figure 4A, Supplementary Figure 3), the T cells derived from the TriVax boosts were more susceptible to PD-1 inhibition (Figure 5B). In addition, both the induction of diabetes and the antitumor efficacy of the vaccine boost administered with TriVax were significantly enhanced by the administration of αPD-L1 mAb (Figures 6 and 7). These data suggest that IL2Cx could circumvent the downstream signaling mediated by PD-1/PD-L1 interactions. One potential target is SHP-2 tyrosine phosphatase, which is recruited and phosphorylated upon PD-1/PD-L1 ligation. Phosphorylated SHP-2 inhibits the phosphoinositide 3-kinase (PI3K), decreasing downstream T cell activation signaling by Akt and Erk1/2, which are crucial for enhancing survival and proliferation. Additionally, signaling through IL-2 has been shown to activate Akt and Erk1/2 through STAT5 activation independently of PI3K pathway [30, 31]. These possibilities yet to be explored, may help explain why T cells from IL2Cx boosted mice can resist PD-1 inhibition regardless of the presence of cell surface PD-1.

In summary, the present studies underline the importance of the selection of appropriate T cell epitopes for vaccine development against tumors. One must be aware of potentially deadly autoimmune consequences in those instances when the vaccination strategies are highly immunogenic and the tumor peptide epitopes are also expressed in vital organs. This word of caution also applies to adoptive T cell therapies, where severe off-target toxicities were observed when the tumor antigen (or a cross-reactive epitope) is also expressed in normal tissues [32, 33]. One way to prevent autoimmune pathology is the use of a tumor-associated antigen not expressed on a vital organ such as the example presented in Figure 8 or a tumor-specific antigen such as mutation-derived neoantigens [34–36].

## MATERIALS AND METHODS

### Mice, cell lines and blood glucose monitoring

Six- to 8-week-old C57BL/6 mice (WT) were obtained from National Cancer Institute/Charles River program. RIP-gp mice were obtained from P. Ohashi (Princess Margaret Cancer Center, University of Toronto, Toronto, Canada) and bred in our facility. B16F10 murine melanoma was obtained from the American Type Culture Collection, Manassas, VA. B16F10 cells transfected with a mini-gene plasmid encoding the LCMV gp33-41 CD8 T cell epitope (B16F10gp33-41) were made by A. Prevost-Blondel, et al., [20], were kindly provided by P. Ohashi and maintained in selection media containing 200 μg/ml G418. Blood glucose levels were measured 2–3 times per week using Accuchek III Glucometers and Chemstrips (Roche) and mice were considered diabetic following 2 consecutive measurements > 250 mg glucose/dl.

### Vaccine components and administration

The following synthetic peptides were used in this study: gp33-41 (KAVYNFATM) representing the minimal H-2Db/Kb restricted CD8 T-cell epitope; gp34-41 (AVYNFATM), the minimal H-2Kb epitope. In addition, the following di-palmitoylated amphiphilic peptide constructs were used: pam-gp33-41 (Pam2-KIIIGIKAVYNFATM); pam-Ova257-264 (Pam2-KMVESIINFEKL); and pam-Trp1.455-463 (Pam2-KMFVTAPDNLGYM). All the peptides were purchased from A&A labs (San Diego, CA) and the identity and the purity (>80%) were determined by high-performance liquid chromatography and mass spectrometry analysis. All peptides were dissolved in dimethyl sulphoxide (DMSO) and 0.1 % Trifluoroacetic acid (TFA) at 20 mg/ml and kept at -80 C. Poly-IC was purchased from InvivoGen (Cat#. tlrl-pic-5). Agonistic αCD40 monoclonal antibody (αCD40 mAb) clone, FGK45.5, was purchased from BioXcell, (Cat# BE0016-2). IL-2 complexes (IL2Cx) were prepared as previously described [37]. Briefly for each dose, 2 μg recombinant murine IL-2 (Biolegend) were incubated with 10 μg of αIL-2 mAb, clone JES6-5H4, BioXcell. For vaccine administration, mice were injected intravenously (i.v.) with BiVax freshly prepared by mixing 100 μg of minimal peptide or 120 μg di-palmitoylated peptide and 50 μg poly-IC in PBS. Two weeks later, mice were boosted with BiVax, BiVax in combination with 100 μg of αCD40 mAb (TriVax) or BiVax in combination with 3 doses of IL2Cx (BiVax/IL2Cx) that were administered i.p. on days 14, 16 and 18. In some instances, CD8 or CD4 T cells were depleted before the booster vaccination by i.p. administration of 2 doses of 500 μg αCD8 mAb (clone 2.43, BioXcell) or 250 μg αCD4 mAb (clone GK1.5, BioXcell), on days 12 (2 days before the booster vaccine) and day14. For PD-L1 blockade, 200 μg of αPD-L1 mAb (clone 10F.9G2, BioXcell) were administered i.p. every 2 days for 3 consecutive doses on days 14, 16 and 18.

### Flow cytometry and the evaluation of immune responses

For measuring antigen-specific CD8 T-cell responses; peripheral blood samples or splenocytes were stained with: PE labeled tetramers gp33-41/H-2Db, gp34-41/H-2Kb, Ova257264/H-2Kb(α1α2)-H-2Db(α3), or Trp1.455-463/H-2Db (all kindly provided by NIH tetramer Core Facility, Emory University, Atlanta, GA). Fluorescent-labeled antibodies were purchased either from eBioscience or Biolegend. Flow cytometry was performed using LSRII Cytometer (BD Biosciences) and data analysis was performed using FlowJo software (version 8.5, Tree Star).

### EliSpot and ELISA

CD8 T cells were purified from splenocytes of vaccinated mice using CD8 positive selection kits (Miltenyi Biotec). For IFNγ EliSpot assays, effector cells were incubated at different numbers (adjusted to the number of tetramer+ T cells), together with 1 × 10^5^ stimulator cells (B16F10, B16F10 pulsed with minimal gp33-41 peptide or B16F10gp33-41 cells). In some experiments, B16F10gp33-41 cells were pre-treated with IFNγ (50 ng/ml) for 48 h. For *in vitro* PD-L1 blockade, 10 μg/ml of αPD-L1 (clone 10F.9G2, BioXcell) was used. ImmunoSpot System (Cellular Technology Ltd, Cleveland, OH) was used to develop and count the cytokine-positive spots. For ELISA, purified CD8 T cells were incubated with different concentrations of minimal gp33-41 or gp34-41 peptides. Two days later, the supernatants were collected and IFNγ production was quantified using an ELISA kit (eBioscience) in accordance with the manufacturer’s protocol.

### Immunofluorescence

Formalin-fixed-paraffin-embedded (FFPE) tissue sections were deparaffinized, antigen retrieval were completed and sections blocked in 2% donkey serum, incubated overnight with rabbit anti-mouse Insulin (4590, 1:100 dilution, Cell signaling), followed by washing in PBS and incubation in donkey anti-rabbit IgG Alexa Fluor 488 (1:200 dilution; Thermo-Fisher Scientific). Next, sections were washed in PBS and mounted in aqueous mounting media with DAPI (ThermoFisher Scientific), and visualized in a confocal microscope (Zeiss LSM 780 Upright Confocal).

### Antitumor experiments

RIP-gp mice were injected subcutaneously with 3 × 10^5^ B16F10gp33-41 cells and tumor growth was monitored every 2 to 3 days in individual tagged mice by measuring 2 opposing diameters with a set of calipers. Results are presented as the mean tumor size (area in mm^2^) ± SD for every treatment group at various time points until the termination of the experiment (usually when tumor size reaches 20 mm diameter).

### Statistical analyses

All experiments were repeated at lest 2 times to ensure reproducibility. Number of mice per experimental group was determined by the minimal number of mice required to obtained statistical significance between the experimental groups. Statistical significance was determined by unpaired Student t tests or one-way ANNOVA. Tumor sizes between 2 populations throughout time were analyzed for significance using 2-way ANOVA. All analyses and graphics were done using Prism 6 software (GraphPad). P values less than 0.05 were considered statistically significant.

## SUPPLEMENTARY MATERIALS FIGURES



## Abbreviations

CTLA-4: cytotoxic T-lymphocyte-associated antigen 4
DCs: dendretic cells
ELISA: enzyme-linked immunosorbent assay
EliSpot: enzyme-Linked immunoSpot assay
Eomes: eomesodermin
gp: glycoprotein
IFNγ: interferon gamma
IL2Cx: Interleukin-2/ αInterleukin-2 mAb complexes
i.p.: intraperitoneal
IU: international unit
i.v.: intravenous
LAG3: leukocyte activation gene
LCMV: lymphocytic choriomeningitis virus
LPS: lipopolysaccharide
Mab: monoclonal antibody
MDSCs: myeloid derived suppressor cells
NK: natural killer cells
NOD: non-obese diabetic mice
Ova: ovalbumin protein
Pam: palmitoylated
PD-1: programmed cell death
PD-L1: programmed cell death ligand
poly-IC: poly-ionosonic poly-cytodylic acid
RIP: rat insulin promoter
s.c.: subcutaneous
Tbet: T box transcription factor
TCR: T cell receptors
TNFα: tumor necrosis factor α
Tregs: regulatory T cells
Trp: tyrosinase related protein

## References

[1] Hodi FS, O'Day SJ, McDermott DF, Weber RW, Sosman JA, Haanen JB, Gonzalez R, Robert C, Schadendorf D, Hassel JC, Akerley W, van den Eertwegh AJ, Lutzky J (2010). Improved survival with ipilimumab in patients with metastatic melanoma. N Engl J Med.

[2] Schmid-Bindert G, Jiang T (2015). First-line nivolumab (anti-PD-1) monotherapy in advanced NSCLC: the story of immune checkpoint inhibitors and “the sorcerers apprentice”. Transl Lung Cancer Res.

[3] Robert C, Ribas A, Wolchok JD, Hodi FS, Hamid O, Kefford R, Weber JS, Joshua AM, Hwu WJ, Gangadhar TC, Patnaik A, Dronca R, Zarour H (2014). Anti-programmed-death-receptor-1 treatment with pembrolizumab in ipilimumab-refractory advanced melanoma: a randomised dose-comparison cohort of a phase 1 trial. Lancet.

[4] Boutros C, Tarhini A, Routier E, Lambotte O, Ladurie FL, Carbonnel F, Izzeddine H, Marabelle A, Champiat S, Berdelou A, Lanoy E, Texier M, Libenciuc C (2016). Safety profiles of anti-CTLA-4 and anti-PD-1 antibodies alone and in combination. Nat Rev Clin Oncol.

[5] June CH, Warshauer JT, Bluestone JA (2017). Is autoimmunity the Achilles' heel of cancer immunotherapy?. Nat Med.

[6] Cho HI, Celis E (2009). Optimized peptide vaccines eliciting extensive CD8 T-cell responses with therapeutic antitumor effects. Cancer Res.

[7] Assudani D, Cho HI, DeVito N, Bradley N, Celis E (2008). *In vivo* expansion, persistence, and function of peptide vaccine-induced CD8 T cells occur independently of CD4 T cells. Cancer Res.

[8] Barrios K, Celis E (2012). TriVax-HPV: an improved peptide-based therapeutic vaccination strategy against human papillomavirus-induced cancers. Cancer Immunol Immunother.

[9] Cho HI, Barrios K, Lee YR, Linowski AK, Celis E (2013). BiVax: a peptide/poly-IC subunit vaccine that mimics an acute infection elicits vast and effective anti-tumor CD8 T-cell responses. Cancer Immunol Immunother.

[10] Cho HI, Reyes-Vargas E, Delgado JC, Celis E (2012). A potent vaccination strategy that circumvents lymphodepletion for effective antitumor adoptive T-cell therapy. Cancer Res.

[11] Ohashi PS, Oehen S, Buerki K, Pircher H, Ohashi CT, Odermatt B, Malissen B, Zinkernagel RM, Hengartner H (1991). Ablation of “tolerance” and induction of diabetes by virus infection in viral antigen transgenic mice. Cell.

[12] Hudrisier D, Oldstone MB, Gairin JE (1997). The signal sequence of lymphocytic choriomeningitis virus contains an immunodominant cytotoxic T cell epitope that is restricted by both H-2D(b) and H-2K(b) molecules. Virology.

[13] Amrani A, Verdaguer J, Serra P, Tafuro S, Tan R, Santamaria P (2000). Progression of autoimmune diabetes driven by avidity maturation of a T-cell population. Nature.

[14] Zhong S, Malecek K, Johnson LA, Yu Z, Vega-Saenz de Miera E, Darvishian F, McGary K, Huang K, Boyer J, Corse E, Shao Y, Rosenberg SA, Restifo NP (2013). T-cell receptor affinity and avidity defines antitumor response and autoimmunity in T-cell immunotherapy. Proc Natl Acad Sci U S A.

[15] Hatton RD, Weaver CT (2003). Immunology. T-bet or not T-bet. Science.

[16] Hersperger AR, Martin JN, Shin LY, Sheth PM, Kovacs CM, Cosma GL, Makedonas G, Pereyra F, Walker BD, Kaul R, Deeks SG, Betts MR (2011). Increased HIV-specific CD8+ T-cell cytotoxic potential in HIV elite controllers is associated with T-bet expression. Blood.

[17] Ribeiro-dos-Santos P, Turnbull EL, Monteiro M, Legrand A, Conrod K, Baalwa J, Pellegrino P, Shaw GM, Williams I, Borrow P, Rocha B (2012). Chronic HIV infection affects the expression of the 2 transcription factors required for CD8 T-cell differentiation into cytolytic effectors. Blood.

[18] Juedes AE, Rodrigo E, Togher L, Glimcher LH, von Herrath MG (2004). T-bet controls autoaggressive CD8 lymphocyte responses in type 1 diabetes. J Exp Med.

[19] Kao C, Oestreich KJ, Paley MA, Crawford A, Angelosanto JM, Ali MA, Intlekofer AM, Boss JM, Reiner SL, Weinmann AS, Wherry EJ (2011). Transcription factor T-bet represses expression of the inhibitory receptor PD-1 and sustains virus-specific CD8+ T cell responses during chronic infection. Nat Immunol.

[20] Prevost-Blondel A, Zimmermann C, Stemmer C, Kulmburg P, Rosenthal FM, Pircher H (1998). Tumor-infiltrating lymphocytes exhibiting high *ex vivo* cytolytic activity fail to prevent murine melanoma tumor growth *in vivo*. J Immunol.

[21] Hixon JA, Blazar BR, Anver MR, Wiltrout RH, Murphy WJ (2001). Antibodies to CD40 induce a lethal cytokine cascade after syngeneic bone marrow transplantation. Biol Blood Marrow Transplant.

[22] Chen L (2004). Co-inhibitory molecules of the B7-CD28 family in the control of T-cell immunity. Nat Rev Immunol.

[23] Dissanayake D, Gronski MA, Lin A, Elford AR, Ohashi PS (2011). Immunological perspective of self versus tumor antigens: insights from the RIP-gp model. Immunol Rev.

[24] Dissanayake D, Murakami K, Tran MD, Elford AR, Millar DG, Ohashi PS (2014). Peptide-pulsed dendritic cells have superior ability to induce immune-mediated tissue destruction compared to peptide with adjuvant. PLoS One.

[25] Lang KS, Recher M, Junt T, Navarini AA, Harris NL, Freigang S, Odermatt B, Conrad C, Ittner LM, Bauer S, Luther SA, Uematsu S, Akira S (2005). Toll-like receptor engagement converts T-cell autoreactivity into overt autoimmune disease. Nat Med.

[26] Millar DG, Garza KM, Odermatt B, Elford AR, Ono N, Li Z, Ohashi PS (2003). Hsp70 promotes antigen-presenting cell function and converts T-cell tolerance to autoimmunity *in vivo*. Nat Med.

[27] Garza KM, Chan SM, Suri R, Nguyen LT, Odermatt B, Schoenberger SP, Ohashi PS (2000). Role of antigen-presenting cells in mediating tolerance and autoimmunity. J Exp Med.

[28] Sultan H, Fesenkova VI, Addis D, Fan AE, Kumai T, Wu J, Salazar AM, Celis E (2017). Designing therapeutic cancer vaccines by mimicking viral infections. Cancer Immunol Immunother.

[29] Wang J, Yoshida T, Nakaki F, Hiai H, Okazaki T, Honjo T (2005). Establishment of NOD-Pdcd1-/- mice as an efficient animal model of type I diabetes. Proc Natl Acad Sci U S A.

[30] Parry RV, Chemnitz JM, Frauwirth KA, Lanfranco AR, Braunstein I, Kobayashi SV, Linsley PS, Thompson CB, Riley JL (2005). CTLA-4 and PD-1 receptors inhibit T-cell activation by distinct mechanisms. Mol Cell Biol.

[31] Yokosuka T, Takamatsu M, Kobayashi-Imanishi W, Hashimoto-Tane A, Azuma M, Saito T (2012). Programmed cell death 1 forms negative costimulatory microclusters that directly inhibit T cell receptor signaling by recruiting phosphatase SHP2. J Exp Med.

[32] Yang JC (2015). Toxicities associated with adoptive T-cell transfer for cancer. Cancer J.

[33] Morgan RA, Yang JC, Kitano M, Dudley ME, Laurencot CM, Rosenberg SA (2010). Case report of a serious adverse event following the administration of T cells transduced with a chimeric antigen receptor recognizing ERBB2. Mol Ther.

[34] Gubin MM, Zhang X, Schuster H, Caron E, Ward JP, Noguchi T, Ivanova Y, Hundal J, Arthur CD, Krebber WJ, Mulder GE, Toebes M, Vesely MD (2014). Checkpoint blockade cancer immunotherapy targets tumour-specific mutant antigens. Nature.

[35] Yadav M, Jhunjhunwala S, Phung QT, Lupardus P, Tanguay J, Bumbaca S, Franci C, Cheung TK, Fritsche J, Weinschenk T, Modrusan Z, Mellman I, Lill JR (2014). Predicting immunogenic tumour mutations by combining mass spectrometry and exome sequencing. Nature.

[36] Cameron BJ, Gerry AB, Dukes J, Harper JV, Kannan V, Bianchi FC, Grand F, Brewer JE, Gupta M, Plesa G, Bossi G, Vuidepot A, Powlesland AS (2013). Identification of a Titin-derived HLA-A1-presented peptide as a cross-reactive target for engineered MAGE A3-directed T cells. Sci Transl Med.

[37] Boyman O, Kovar M, Rubinstein MP, Surh CD, Sprent J (2006). Selective stimulation of T cell subsets with antibody-cytokine immune complexes. Science.

