# Supplementary material for: Effective antitumor peptide vaccines can induce severe autoimmune pathology
Source: Oncotarget. 2017 Jul 29;8(41):70317–31. doi: 10.18632/oncotarget.19688 (PMC5642557; doi:10.18632/oncotarget.19688)
Supplement: Supplementary file 1 [file oncotarget-08-70317-s001.pdf]

Effective antitumor peptide vaccines can induce severe autoimmune pathology

SUPPLEMENTARY MATERIALS

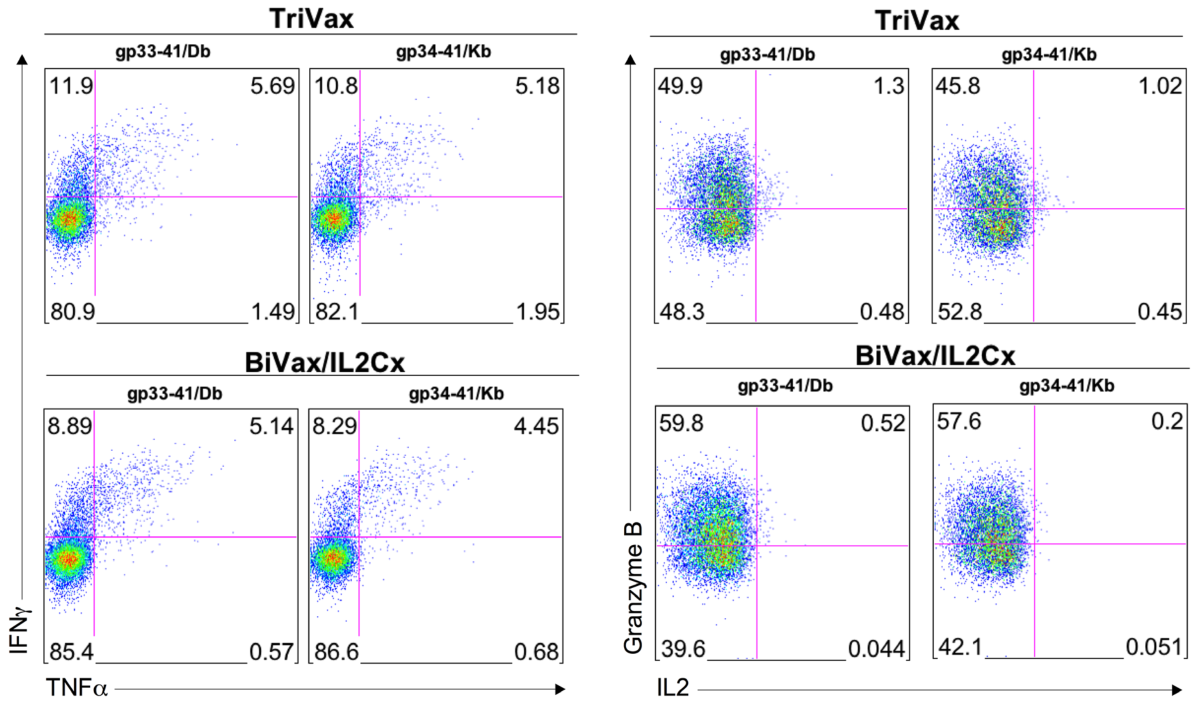

Supplementary Figure 1: Intracellular cytokine staining. Representative FACS dot plots showing the intracellular cytokines staining described in Figure 2D.

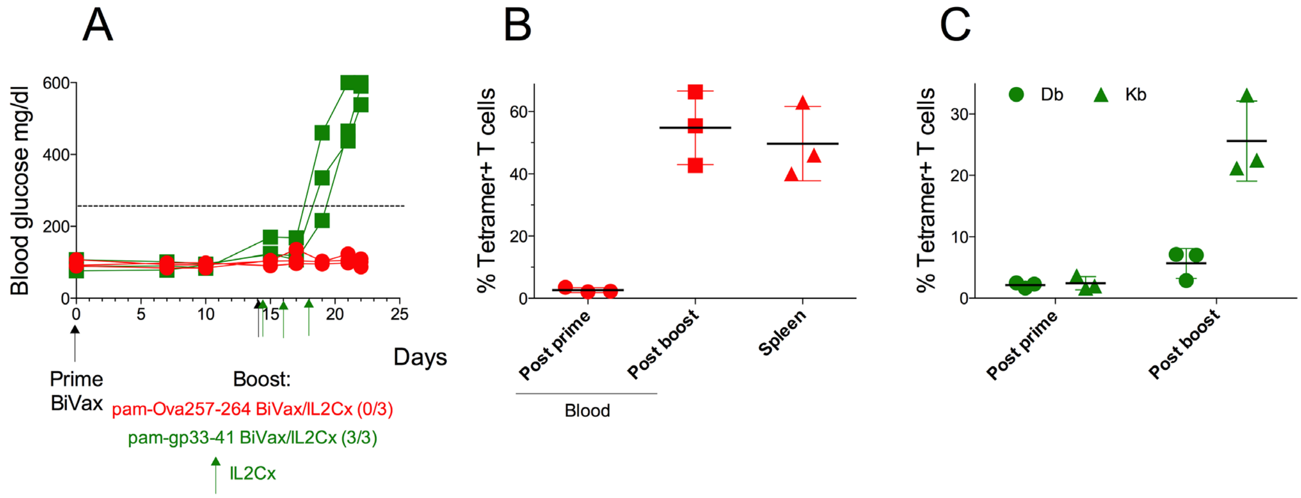

Supplementary Figure 2: PamOva257-264 BiVax/IL2Cx does not induce diabetes. RIP-gp mice were primed with pam-gp33-41 BiVax or pam-Ova257-264 BiVax. Fourteen days later, mice were boosted with pam-gp33-41or pam-Ova257-264 BiVax plus IL2Cx on days 14, 16 and 18. **(A)** Blood glucose levels in individual mice (each symbol) in each group. **(B)** The percentage of Ova257-264 specific CD8 T cells in blood and spleen of the pam-Ova257-264 immunized mice. **(C)** The percentage of Kb and Db specific CD8 T cells in blood of pam-gp33-41 immunized mice. Results are presented for individual mice (each symbol) with the mean ± SD for each group.

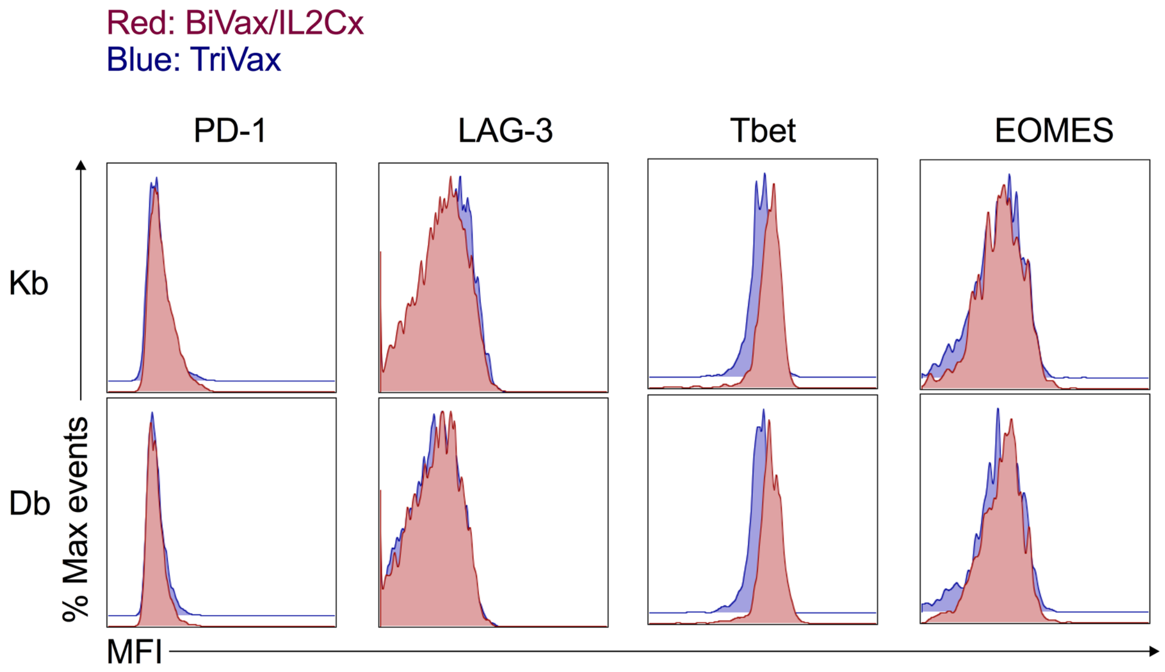

Supplementary Figure 3: PD-1. LAG-3 and transcription factor expression levels. Representative histogram plots showing the expression level of PD-1, LAG-3, Tbet and Eomes described in Figure 4A-4D.

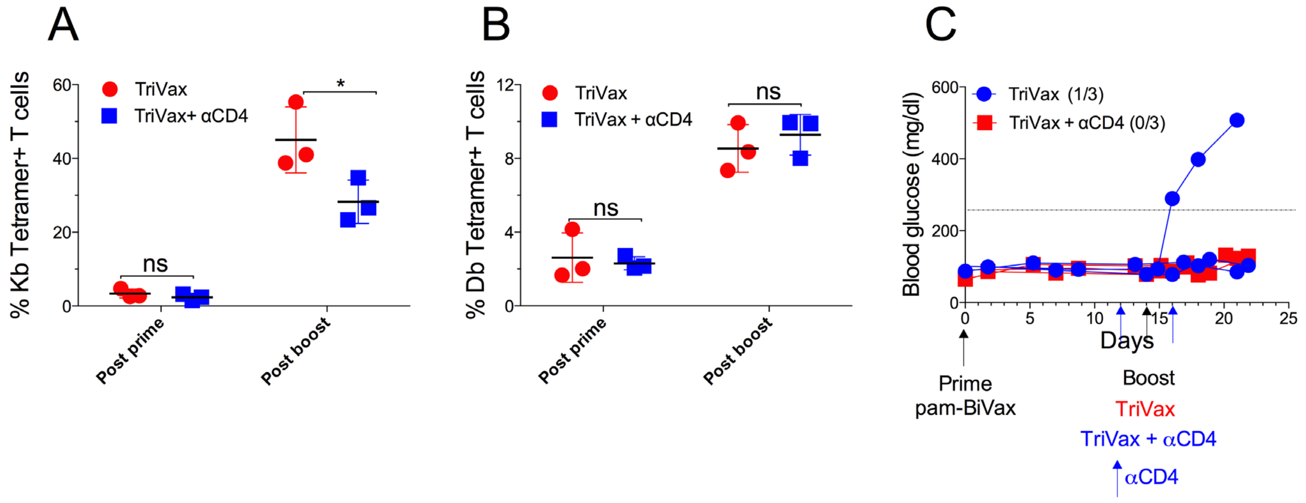

Supplementary Figure 4: CD4 T cell (Treg) depletion in TriVax boost does not increase diabetes. RIP-gp mice were primed with pam-gp33-41BiVax and 14 days later they received TriVax boosts with or without CD4 T cell depletion (αCD4 mAb 200 µg/mouse i.p. on days 12 and 14). The percentage of Kb **(A)** and Db **(B)** tetramer+ CD8 T cells in blood after prime and after boost. **(C)** Blood glucose levels in individual mice (each symbol) for each group. Results presented for individual mice (each symbol) with the mean ± SD for each group. (*p<0.05, ns: not significant).
